# Optical simulation of a Popescu-Rohrlich Box

**DOI:** 10.1038/srep28351

**Published:** 2016-06-22

**Authors:** Wen-Jing Chu, Xiao-Lan Zong, Ming Yang, Guo-Zhu Pan, Zhuo-Liang Cao

**Affiliations:** 1 School of Physics & Material Science, Anhui University, Hefei 230601, China; 2 School of Material and Chemical Engineering, West Anhui university, Lu’an, 237012, China; 3 Institute for Quantum Control and Quantum Information, School of Electronic and Information Engineering, Hefei Normal University, Hefei 230601, China

## Abstract

It is well known that the fair-sampling loophole in Bell test opened by the selection of the state to be measured can lead to post-quantum correlations. In this paper, we make the selection of the results after measurement, which opens the fair- sampling loophole too, and thus can lead to post-quantum correlations. This kind of result-selection loophole can be realized by pre- and post-selection processes within the “two-state vector formalism”, and a physical simulation of Popescu-Rohrlich (PR) box is designed in linear optical system. The probability distribution of the PR has a maximal CHSH value 4, i.e. it can maximally violate CHSH inequality. Because the “two-state vector formalism” violates the information causality, it opens the locality loophole too, which means that this kind of results selection within “two-state vector formalism” leads to both fair- sampling loophole and locality loophole, so we call it a comprehensive loophole in Bell test. The comprehensive loophole opened by the results selection within “two-state vector formalism” may be another possible explanation of why post-quantum correlations are incompatible with quantum mechanics and seem not to exist in nature.

In 1935, Einstein, Podolsky and Rosen claimed that quantum wave function does not provide a complete description of physical reality, which is called EPR paradox^1^. Based on EPR paradox and hidden variable theory, Bell quantitatively analyzed and put forward the Bell inequality in a seminal paper in 1964^2^. More precisely, the hidden variable theory is expressed in mathematics, which reveals that spatially separated quantum systems can have strong correlations. This kind of correlation is known as nonlocality, and it plays a crucial role in quantum information theory, such as nonlocal computation^3^. Meanwhile, Bell theory provides a significant criterion for the experimentalists to prove the validity of quantum mechanics, and the corresponding experiments verified the nonlocality property of quantum mechanics^4^,^5^,^6^.

From John Bell’s original inequality, John Clauser, Michael Horne, Abner Shimony, and Richard Holt derived a new inequality—CHSH inequality—in a much-cited paper published in 1969^7^. The maximum violation of CHSH inequality can reach 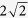 in quantum mechanics domain, i.e. the so-called Tsirelson’s bound^8^, rather than the maximum value 2 in classical domain. Up to now, most of the previous Bell inequality test experiments suffer from the following three loopholes, i.e. the locality loophole (or communication loophole), the freedom-of-choice loophole, and the fair-sampling loophole (or detection loophole). The fact that the measurement choice on one subsystem may influence the outcome of the other (and vice-versa) opens the locality loophole. In a Bell test, the two users must be free to choose random measurement choices that are physically independent of one another and of any property of the particles, otherwise, there comes the freedom-of-choice loophole. The detection efficiency must be independent of the measurement settings, i.e. the sample of detected pairs provides a fair statistical sample of all the pairs. If this is not true, it opens the fair-sampling loophole (or detection loophole). The results of the Bell test experiments with any one of these three loopholes only can be accepted with some assumptions. Very recently, Bell tests that close the most significant two loopholes simultaneously have been reported^9^,^10^,^11^,^12^.

Although loopholes have negative effects on Bell test, they play constructive roles in simulating post-quantum correlations whose violations of Bell inequality surpass the so-called Tsirelson’s bound. The most typical representative of this kind of correlations is the famous Popescu and Rohrlich (PR) correlation. Popescu and Rohrlich showed that it is possible to construct various causality satisfying models, where the violation of CHSH inequality can exceed the quantum mechanical bound and reach the algebraic maximal value 4^13^. The nonlocality revealed by the violation of Bell’s inequality can be described by a correlation box shared between two parties. The boxes with the algebraic maximal violation 4 of CHSH inequality are termed PR boxes. Even though previous researches^14^,^15^,^16^,^17^ suggested that these post-quantum correlations cannot be implemented by classical or quantum systems, they can be simulated by exploiting the loopholes in a Bell test. Obvious violations of Bell inequality beyond Tsirelson’s bound caused by the fair-sampling loophole (or detection loophole) have been observed in experiments where one of the entangled photons is measured and amplified^18^ or re-generated^18^,^19^, or we have the knowledge of the states being measured^20^. The loss-induced fair-sampling loophole (or detection loophole) can lead to a violation of Bell inequality beyond Tsirelson’s bound too^21^,^22^,^23^,^24^,^25^. Among these studies, the fair-sampling loophole is opened by the selection of states to be measured. Actually, the fair-sampling loophole is still open if we select the results after measurement. In other words, it is still possible to simulate post-quantum correlations via post-select the measurements results. Cabello showed this possibility by simulating bipartite correlations beyond Tsirelson’s bound via appropriately post-selecting two qubits of a three-qubit GHZ state system^26^, and Chen *et al*. observed this kind of supercorrelations in optical system experimentally^27^. But this kind of simulation of post-quantum correlation must make use of tripartite state, which obviously limits its persuasiveness. If this kind of selection is done directly on the two subsystems in a bipartite entangled state, the effect of the result-selection induced fair sampling loophole can be shown more obviously. Marcovitch *et al*. showed that it can be done within “two-state vector formalism”, namely, a state described by “two-state vector formalism” can exhibit a strong violation of CHSH inequality, which can exceed Tsirelson’s bound and even reach the algebraic maximal value (4)^28^. Here, the measurement is done on all the samples, and the post-selection is only done after measurement, which is more in line with Bell theory than the case with the selection of the states before measurement. The “two-state vector formalism” is a new concept defined by Yakir Aharonov and Lev Vaidman, which is a complete description of a quantum system at a given time based on the results of experiments performed both before and after this time^29^. In addition, because the “two-state vector formalism” violates the information causality^25^,^30^, it opens the locality loophole too , which makes this kind of result-selection induced loophole a comprehensive loophole (including both locality and fair-sampling loopholes) in Bell test. Besides information causality^25^,^30^, the comprehensive loophole opened by the result selection within “two-state vector formalism” is another possible explanation of why post-quantum correlations are incompatible with quantum mechanics and seem not to exist in nature.

So, in this paper, we will propose a physical scheme for simulating PR correlations by using the comprehensive loophole opened by the result selection within “two-state vector formalism”. In linear optical system, a PR correlation can be simulated by appropriately pre-selecting photon ensemble to be measured and post-selecting the measurement results. Because all the optical elements used here are very common ones, the physical scheme proposed here is feasible.

## Results

**Figure 1:**
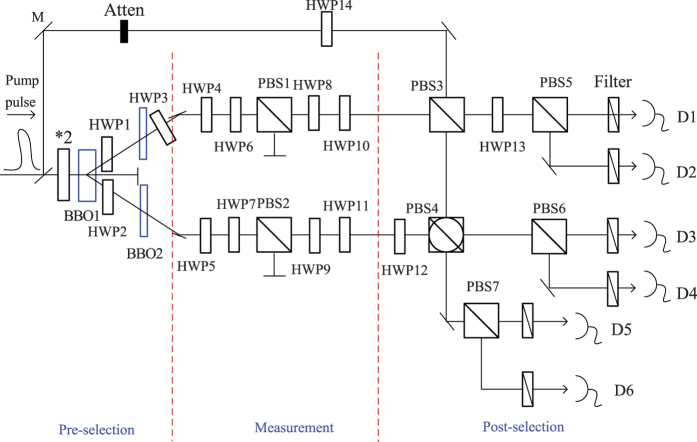
The optical simulation of a PR box. BBO1 is a nonlinear crystal (beta-barium borate), and BBO2 is half the thickness of BBO1. M is a mirror, HWP denotes a half wave plate. PBSi (i = 1, 2, 3, 5, 6, 7) denotes a general polarizing beam splitter, which transmits horizontally polarized photons and reflects vertically polarized photons. PBS4 is a rotated polarizing beam splitter, which transmits the photons in state 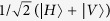 and reflects the photons in state 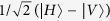. D1, D2, D3, D4, D5, D6 are single-photon detectors. Sapphire laser were frequency doubled (*2) to provide UV pulses , which are used to pump BBO1 crystal. The results are registered through a three-port coincidence circuit (D2, D3 and D5) with a coincidence window.

**Figure 2:**
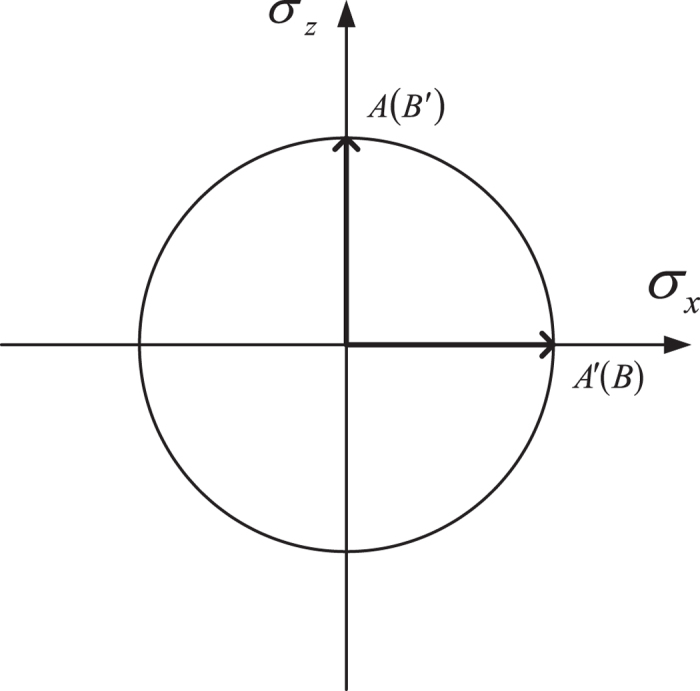
The schematic diagram of the observables of Alice and Bob.

In this section, we will design an optical setup, through which the probability distribution obtained from the ideal measurements on a pre- and post-selected ensemble of polarization entangled photon pairs is exactly a PR probability distribution. As depicted in Fig. 1, the whole setup includes three stages: the pre-selection, the measurement and the post-selection. A piece of the type-2 BBO crystal (BBO1) is pumped by a femtosecond laser pulse, producing a pair of entangled photons in the state 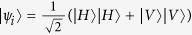 with a half wave plate (HWP3) set at 45°, and the BBO2, being half the thickness of BBO1, is a compensation for the longitudinal walk-off with two HWPs (1, 2) set at 45°^31^. The entangled photons are produced coherently along the entire length of the crystal, which induces the longitudinal walk-off between two different polarizations^32^, so the relative time delay equals *L*/2(1/*u*_*v*_ − 1/*u*_*h*_) (*L* is the crystal length, and *u*_*v*_ and *u*_*h*_ are the vertical and horizontal polarization velocities). The two HWPs (HWP1 and HWP2) rotate the polarization of the beams by 90°, and thus the retardations of the *h* and the *v* components are exchanged, so it can be restored by the BBO2. Here finishes the pre-selection process with the initial state 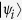 being chosen, and the next stage is the ideal measurement process. Alice (Bob) will carry out projective measurements by using polarizing beam splitters-PBS1(PBS2), HWP4(HWP5) and HWP10(HWP11). PBS1 with HWPs (HWP4 and HWP10) setting at 0° can transmit the photons in state |*H*〉 and reflect the photons in state |*V*〉, and PBS1 with HWPs (HWP4 and HWP10) setting at 22.5° can transmit the photons in state 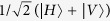 and reflect the photons in state 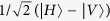. So the angle of HWP10 is always equal to that of HWP4, and similarly the angle of HWP11 is always equal to that of HWP5. More precisely, the two possible angles of HWP4(HWP5) and HWP10(HWP11) correspond to two different measurement directions as specified in Fig. 2. That is to say, the four possible angle combinations for the wave plates HWP4(HWP10) and HWP5(HWP11) have a one-to-one relation to four different inputs (*x*, *y*) = (0, 0), (0, 1), (1, 0), (1, 1) of a PR box. To simplify this setup, we have used four HWPs (HWP6, HWP7, HWP8 and HWP9) to simulate different measurement results. The PBS 1 with HWP6 and HWP8 both set at 0° can transmit |*H*〉, while it can transmit |*V*〉 if HWP6 and HWP8 are both set at 45°. So the angle of HWP8 is equal to that of HWP6, and similarly the angle of HWP9 is equal to that of HWP7. The last stage is to post-select those photons whose states are 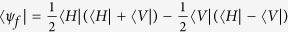, which can be implemented by a controlled-NOT (CNOT) gate, two HWPs (HWP12 and HWP13) both set at 22.5° and two PBSs (PBS5 and PBS6). A single auxiliary photon and three PBSs (PBS3, PBS4 and PBS7) constitute a successful CNOT gate^33^, and more specifically, the detection of a single photon D5 projects the output in the control and target modes into the desired CNOT transform of the input. To generate a good interference between the auxiliary photon and one of the entangled photons on PBS3, the auxiliary photon is generated from the pumping pulse too, and a HWP14 set at 22.5° prepares it in the state 
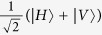
. The detailed implementation setup is shown in Fig. 1.

For one specific angle combination of HWP4 and HWP5, the input of the box is fixed, i.e. (*x*, *y*). A click on D5 indicates the success of the CNOT gate, so a three-photon (D2, D3, D5) coincidence measurement means that the post-selection succeeds. That is, the result of the ideal measurement between pre- and post-selection processes is |*H*〉 |*H*〉 when all the HWPs (HWP4, HWP5, HWP6, HWP7, HWP8, HWP9, HWP10 and HWP11) are set at 0°, so the output of the box is *a* = 0, *b* = 0. The count rate of this coincidence measurement is equal to *p* (00|01). To measure other components, such as *p* (10|01), we can set the angle of the HWP6 and HWP8 at 45°, which flips the value of *a* with *b* unchanged. From the sixteen HWPs settings listed in Table 1, we can get sixteen probabilities. List these sixteen probabilities in a 4 × 4 matrix, we can get an exact probability distribution of a PR box. In this sense, we say this setup can simulate the PR box.

## Conclusion

Based on the results selection after measurement within “two-state vector formalism”, an optical setup is proposed for simulating a PR box. The probabilities of obtaining different states for the ideal measurements between the pre- and post-selection processes are exactly equal to those of a PR box. In our proposal, these probabilities can be easily read from the coincidence rate of a three-photon coincidence measurement. The results selection after measurement opens an obvious fair-sampling loophole here, and the violation of information causality caused by “two-state vector formalism” opens the locality loophole too, which makes this kind of result-selection induced loophole a comprehensive loophole. Thus, besides information causality, the comprehensive loophole opened by the result selection within “two-state vector formalism” is another possible explanation of why post-quantum correlations are incompatible with quantum mechanics and seem not to exist in nature.

In addition, all the elements used here, such as BBO crystals, HWPs, PBSs and photon detectors, are very common elements within the current experimental quantum information technology, so the current proposal can be realized in Lab. Hope the current proposal can ignite further experimental investigations on post-quantum correlation.

## Methods

The correlations beyond Tsirelson’s bound can be described by a black box in nonsignaling theory^13^. The box is shared by two space-like separated users Alice and Bob who will give the box inputs *x*, *y*, respectively. Then they will get the corresponding outputs *a* and *b* with probability *p* (*ab* | *xy*), where *x*, *a*, *y*, *b* ∈ {0, 1}. Hence the joint probability distribution is expressed as
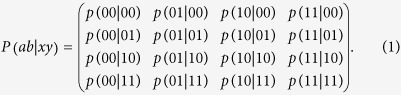


The elements of the probability distribution matrix meet the nonnegativity condition,
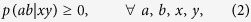
and the normalization condition, too
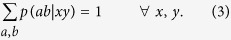


Classical communications are forbidden throughout the measurement process, that is to say the input and output of one user does not affect those of the other’s. Thus, the marginal probabilities *p* (*a*|*x*) and *p* (*b*|*y*) are independent of *y* and *x*, respectively:
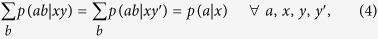

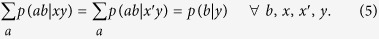


The discussions in ref. 13 show that the correlations of the black boxes defined above can surpass the Tsirelson’s bound and a typical box with the post-quantum correlation is the PR box whose CHSH value reaches the algebraic maximum 4:
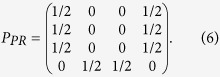


But, it is well known that no quantum state can violate the CHSH inequality beyond Tsirelson’s bound 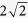
7, so it is not possible to realize a post-quantum correlation with a quantum state. One has to find new methods to simulate post-quantum correlations, especially the maximally post-quantum correlation 4 revealed by a PR box. The key point here is to find the rules to get the PR probability distribution in Eq. (6). Marcovitch *et al*. showed that under “two-state vector formalism” one can get probability distributions with their violations of the CHSH inequality beyond Tsirelson’s bound, and even the algebraic maximal violation value (4) can be achieved^28^.

In standard quantum theory, the state of a quantum system is determined by the system’s past. Aharonov and Vaidman provided an alternative description of quantum systems—“two-state vector formalism”^29^. This formalism gives a more complete description of a quantum system than the standard quantum theory, i.e. the description of a quantum system at a given time not only depends on the results of experiments performed before this time but is based also on the results of experiments performed after this time. For instance, there is an ensemble of quantum systems whose states are pre- and post-selected by the following initial and final states:
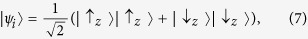

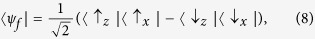
respectively. |↑_*k*_〉, |↓_*k*_〉 are the eigenvectors of Pauli operators *σ*_*k*_ (*k* = *x*, *y*, *z*) respectively. An ideal measurement of an observable *A*(*B*) will be carried out by Alice (Bob) on the ensemble at intermediate time *t*_*i*_ < *t* < *t*_*f*_, and the probability of getting *α*(*β*) is given by^29^
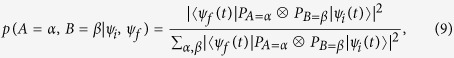
where *P*_*A*=*α*_, *P*_*B*=*β*_ are the projections onto the space of eigenvalues *A* = *α*, *B* = *β*. If each user only has two choices of observables, i.e. there are only two observables *A*, *A*′ for Alice, and *B*, *B*′ for Bob, the joint probability *p*(*αβ*|*AB*) of the event where Alice gets eigenvalue *α* by measuring observable *A* and Bob gets *β* on *B* exactly simulates the joint probability *p* (*ab*|*xy*) in Eq. (1). Here, Alice’s (Bob’s) two possible observalbes *A*, *A*′(*B*, *B*′) are in a one-to-one correspondence with the two values (0, 1) for the input *x*(*y*) of the box. Being the eigenvalue of the observable *A*(*B*) in two-dimensional Hilbert space, *α*(*β*) only has two possible values +1 and −1, so the two eigenvalues +1 and −1 of an observable are in a one-to-one correspondence with the two possible output values 0 and 1, respectively, of the box. For instance, the measured observables are *A*′ and *B*, and the corresponding output values are *α* = −1 and *β* = 1, respectively, then *p* (*α*, *β*|*A*′*B*) = *p*(10|10). The corresponding correlation function of CHSH inequality is *C*(*A*′, *B*) = *p* (1, 1|*A*′*B*) + *p*(−1, −1|*A*′*B*) − *p* (1, −1|*A*′*B*) − *p* (−1, 1|*A*′*B*). If Alice and Bob perform measurements along the *z* and *x* axes (as depicted in Fig. 2) on the above pre- and post-selected ensembles, they can get *p* (00|00) = *p* (11|00) = *p* (00|01) = *p* (11|01) = *p* (00|10) = *p* (11|10) = *p* (01|11) = *p* (10|11) = 1/2 using the Eq. (9), and the joint probabilities for the other combinations are zero. That is to say, the PR box expressed in Eq. (6) is simulated. To be specific, let’s demonstrate an example in optical system as shown in Fig. 1. For Alice, the two possible inputs (*x* = 0, 1) of the box corresponds to two possible observables *σ*_*z*_ and *σ*_*x*_, respectively, and, on the contrary, the two possible inputs (*y* = 0, 1) of the box corresponds to two possible observables *σ*_*x*_ and *σ*_*z*_, respectively, for Bob. For both users, they only have two possible measurement results +1(|↑〉), −1(|↓〉), which corresponds to the two values 0 and 1 of the box output *a*(*b*), respectively. So *p* (01|00) denotes the probability of the event where Alice measures *σ*_*z*_ and gets |↑〉 with Bob getting |↓〉 in measuring *σ*_*x*_ at intermediate time *t*_*i*_ < *t* < *t*_*f*_:
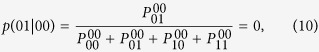
where,
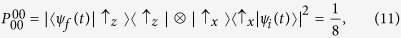

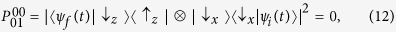

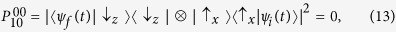

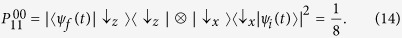


Similarly, we can obtain *p* (10|00) = *p* (01|01) = *p* (10|01) = *p* (01|10) = *p* (10|10) = *p*(00|11) = *p* (11|11) = 0 and *p* (00|00) = *p* (11|00) = *p* (00|01) = *p* (11|01) = *p* (00|10) = *p* (11|10) = *p* (01|11) = *p* (10|11) = 1/2, and all these probabilities constitute a probability distribution matrix:
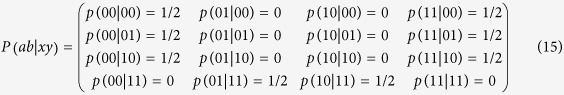


It is easy to find that the probability distribution matrix in Eq. (15) is exactly the same as the matrix in Eq. (6), which means that the setup proposed here can exactly simulate a PR box.

Here, the SPDC equipments (BBO1, two BBO2s, HWP1, HWP2 and HWP3) can pre-select the state 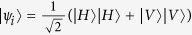, and then, adjusting the angles of eight HWPs (HWP4-11) can complete the measurement with two PBSs (PBS1 and PBS2). At last, the CNOT gate (an auxiliary photon, PBS3, PBS4 and PBS7), two HWPs (HWP12 and HWP13), two PBSs (PBS5 and PBS6) and six detectors can post-select the state 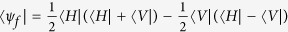. For example, if we want to get the *p* (01|00), set the HWP5 and HWP11 at 22.5°, HWP7 and HWP9 at 45° and HWP4, HWP6, HWP8, HWP10 at 0°, where HWP4 set at 0° and 22.5° corresponds to the two possible input values of the box’s upper side 0 and 1, respectively, i.e. the two possible measurement bases, and, on the contrary HWP5 set at 22.5° and 0° corresponds to the two possible input values of the box’s lower side 0 and 1, respectively. HWP6(HWP 7) set at 0° and 45° corresponds to the two possible output values of the box’s upper (lower) side 0 and 1, respectively, i.e. two possible measurement results on the photons. The initial state 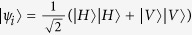 is generated by the SPDC, which will be transformed into 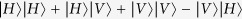 after unitary transformations (HWP4-7). Only the component 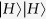 can transit the PBSs (PBS1, 2), and thus the result state 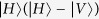 we want can be achieved after four other HWPs (HWP8-11). The result state 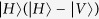 of the measurement stage can be reexpressed in terms of the entangled basis, one basis state of which is the final state in Eq. (8). To discriminate (post-select) this final state from other three basis states, a CNOT gate (an auxiliary photon, PBS3, PBS4, PBS7, D5 and D6)and two local operations (HWP12 and HWP13) are introduced to transform the joint entangled basis measurement into the product basis measurement. The post-selection process is just to pick up the measurement result corresponding to the final state 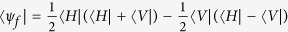. After these transformations, the finial state 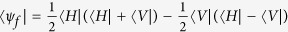 evolves into 〈*H*| 〈*H*|, so the results we want are registered through a three-port coincidence circuit (D2, D3 and D5), and this three-photon coincidence rate is just the probability *p* (01|00). By adjusting the HWP 6 and HWP 7 according to the Table 1, we can get other three probabilities for the input 00, *p* (00|00), *p* (10|00) and *p* (11|00). By adjusting HWP4 and HWP5, the input can be changed. In the same way, we can get the sixteen probabilities for a PR box by adjusting the HWPs according to the Table 1.

## Additional Information

**How to cite this article**: Chu, W.-J. *et al*. Optical simulation of a Popescu-Rohrlich Box. *Sci. Rep.* **6**, 28351; doi: 10.1038/srep28351 (2016).

## Figures and Tables

**Table 1:** The angle settings of HWPs.

*p* (*ab*|*xy*) HWPs	HWP4 (10)	HWP5 (11)	HWP6 (8)	HWP7 (9)
*p* (00|00)	0°	22.5°	0°	0°
*p* (01|00)	0°	22.5°	0°	45°
*p* (10|00)	0°	22.5°	45°	0°
*p* (11|00)	0°	22.5°	45°	45°
*p* (00|01)	0°	0°	0°	0°
*p* (01|01)	0°	0°	0°	45°
*p* (10|01)	0°	0°	45°	0°
*p* (11|01)	0°	0°	45°	45°
*p* (00|10)	22.5°	22.5°	0°	0°
*p* (01|10)	22.5°	22.5°	0°	45°
*p* (10|10)	22.5°	22.5°	45°	0°
*p* (11|10)	22.5°	22.5°	45°	45°
*p* (00|11)	22.5°	0°	0°	0°
*p* (01|11)	22.5°	0°	0°	45°
*p* (10|11)	22.5°	0°	45°	0°
*p* (11|11)	22.5°	0°	45°	45°
